# Sexually transmitted infections in women deprived of liberty in Roraima, Brazil

**DOI:** 10.11606/s1518-8787.2020054002207

**Published:** 2020-10-28

**Authors:** Maria Soledade Garcia Benedetti, Audrey Stella Akemi Nogami, Beatriz Belo da Costa, Herbert Iago Feitosa da Fonsêca, Igor dos Santos Costa, Itallo de Souza Almeida, Luana de Miranda, Matheus Mychael Mazzaro Conchy, Renan da Silva Bentes, Suzani Naomi Higa, Tháles de Souza Israel, Allex Jardim da Fonseca

**Affiliations:** I Universidade Federal de Roraima Centro de Ciências da Saúde Boa VistaRR Brasil Universidade Federal de Roraima . Centro de Ciências da Saúde . Curso de Medicina. Boa Vista , RR , Brasil

**Keywords:** Women, Prisons, Sexually Transmitted Diseases, epidemiology, Risk Factors, Health Status Disparities

## Abstract

**OBJECTIVE:**

To evaluate the prevalence of HIV, syphilis and hepatitis B infection among women deprived of liberty in the state of Roraima, Brazil, and its correlation with perceptions, knowledge and behavioral factors.

**METHOD:**

This is a cross-sectional study, with simple systematic sampling, conducted at the Public Female Prison in Boa Vista, State of Roraima, in 2017. A total of 168 inmates (93.8% of the population) were evaluated by in-person interviews and rapid tests.

**RESULTS:**

The prevalence of sexually transmitted infections (STI) was 20.2%, being 4.7% HIV, 15.5% syphilis, and 0.0% hepatitis B. Multivariate analysis confirmed as risk factors for acquiring an STI: being over 30 years of age [adjusted odds ratio (OR): 2.57; 95%CI 1.03–6.40); low schooling (adjusted OR: 2.77; 95%CI 1.08–5.05); little knowledge about condom use (adjusted OR: 2.37; 95%CI 1.01–7.31); and believing that there is no risk of contracting syphilis (adjusted OR: 2.36; 95%CI 1.08–6.50).

**CONCLUSION:**

The population deprived of liberty is a group of highly vulnerable to STI. The high prevalence of these infections can be explained by knowledge deficits on the subject, distorted perceptions and conditions peculiar to imprisonment, which result in risky behavior. We emphasize the need to implement educational programs for preventing, diagnosing and treating STI for this population.

## INTRODUCTION

The onset of acquired immunodeficiency syndrome (AIDS) in prisons occurred concomitantly with its emergence in the community ^1^ . The first AIDS cases in prisons were recorded in the early 1980s in the United States, in the cities of New York and New Jersey ^2^ . Since then, several studies have warned that the prevalence of HIV and other sexually transmitted infections (STI) among convicted prisoners is significantly higher than in the general population, in which risk behaviors prior to and during prison may contribute to ^3^ .

In Brazil, specific studies conducted in female prisons in some regions revealed a high prevalence of STI in incarcerated women: from 0.0% to 26% for HIV ^4 , 5^ , 2.2% to 22.8% for syphilis ^6 , 7^ , and 3.8% to 26.4% for hepatitis B ^4 , 8^ . However, the factors associated with a higher risk of contracting HIV or STI in this particular population, especially in female prisons, where the risk of STI transmission among inmates is significantly lower compared with male inmates, are unclear in the medical literature. Additionally, we cannot extrapolate the knowledge of factors associated with STI transmission in the general population to the prison population due to the peculiar condition of deprivation of liberty. Incarceration can have a multifactorial influence in these women’s vulnerability, not only because they have less access to knowledge about prevention and transmission mechanisms, but also because of exposure to sexual violence, distortion of risk perceptions or simply restricted access to condoms and consultations with health professionals.

Studies on HIV/STI epidemiology in prison populations are still scarce, especially in northern Brazil. Considering the alarming evidence of the vulnerability of incarcerated women, the prison can be considered a place of special scientific interest, besides being opportune for diagnosing and treating infections. This study aims to evaluate the prevalence of HIV, syphilis and hepatitis B infection in women deprived of liberty in the state of Roraima and its correlation with knowledge, perception, behavior and sociodemographic factors.

## METHODS

### Study design

This is a cross-sectional study designed to evaluate the seroprevalence of HIV/STI infection among women deprived of liberty in the state of Roraima, using rapid tests and a questionnaire addressing the factors associated with the infection, conducted in 2017.

### Population

Roraima is the northernmost state of the Brazilian Legal Amazon and has a population of about 500,000 inhabitants, as estimated by the Brazilian Institute of Geography and Statistics (IBGE) in 2017. It stands out for having two international borders (with Venezuela and Guyana) and for harboring the largest indigenous population in Brazil (approximately 15% of the state’s population).

The research was conducted at the Public Female Prison in Boa Vista (capital), which is the only female penal establishment in the state and housed 179 inmates at the time of the study. Roraima occupies the penultimate place in the national ranking of the number of women deprived of liberty, housing 0.4% of the incarcerated female population in the country; however, it has the fifth highest imprisonment rate in Brazil, of 67 per 100,000 women. The occupancy rate of the state’s female prison system is 228%, almost double the national average for female units. Women constitute 7.2% of the state’s prison population, and almost half of them (48%) are convicted; the others are sentenced to the closed regime (13%), the semi-open regime (12%) and the open regime (28%) ^9^ .

The study took place in October and November 2017, before the massive migration of Venezuelan refugees to Brazil via Roraima.

### Sample and Sampling

To calculate the sample size, we estimated the prevalence of STI in the target population at 20%, based on similar studies. Considering a 95% confidence interval and acceptable error of 5%, we obtained a minimum sample size of 254 inmates. After adjusting the sample target for a small population (n = 179), we obtained a minimum sample size of 146 participants. Considering sample losses and rejection of participation in tests of up to 10%, the final sample target comprised 161 women. The sampling method was simple systematic: all inmates from all cells were invited to participate, without selection and consecutively, according to the cell arrangement in the prison unit blocks.

### Research Procedure

All inmates were individually invited to participate in the study, escorted by prison guards, in their cells. Invitation accepted, the inmate was transferred to a private room where, after extensive clarification, she formally registered her consent. Each participant, anonymously and privately, answered a questionnaire, whose first part was applied by the researcher (face-to-face interview), addressing demographic data, knowledge and perception about HIV/STI. The second part of the questionnaire was self-administered, addressing issues of sexual exposure and drug use, and should be secretly put in a sealed box. In this step, illiterate participants were assisted by the research physicians. The semi-structured questionnaire was adapted from the study by Miranda et al. ^10^ .

Subsequently, we invited the participants to undergo the rapid tests for HIV, which uses immunochromatographic technology, by a qualitative assay, to detect HIV-1/2 specific antibodies (TR DPP ^®^ HIV-1/2, Bio-Manguinhos, Rio de Janeiro, Brazil); syphilis (Alere Syphilis), via the qualitative detection of IgG, IgM and IgA antibodies against *Treponema pallidum* , characterized as a treponemal test (Importador Alere S/A, São Paulo, Brazil); and hepatitis B (Vikia ^®^ HBsAg, BioMérieux SA, France), via the qualitative detection of HBs antigen.

A digital puncture was performed with sterile lancet, in addition to pre- and post-test counseling, and the result was reported within 30 minutes, individually. Non-reactive results were considered negative. In cases of reagent or inconclusive HIV outcome, we retested the participant using the Bioeasy rapid or immunochromatographic test for qualitative detection of HIV-1 specific antibodies (IgG, IgM and IgA isotopes), including subtype O, and HIV-2, simultaneously. If positive, the result was considered confirmed as HIV. For hepatitis B, a positive rapid test is confirmatory and, for syphilis, the rapid test is serological screening. All positive cases were referred to specialized service.

### Data analysis

The data of each participant were stored and analyzed using the Epi Info ^TM^ software, version 7.2.2.6. We calculated the prevalence of HIV, syphilis and hepatitis B in the sample, and conducted a descriptive analysis of sociodemographic characteristics, nature of conviction, type of regimen, sentence length and incarceration length (in months), using measures of central tendency for continuous variables and percentages for categorical variables.

The chi-square test was used to compare differences in proportions of categorical variables, adopting a significance level of 5%. Odds ratio (OR) and 95% confidence interval (95%CI) were calculated in univariate analysis, with OR adjusted in multivariate analysis by logistic regression. The criterion for selecting explanatory variables for entry into the multivariate analysis was the critical value of p < 0.15 in the univariate analysis.

### Ethical aspects

The study was approved by the Research Ethics Committee involving human beings of the Universidade Federal de Roraima (UFRR) – Opinion No. 1,797,708 and CAAE 44535315.3.0000.5302. It was also formally authorized by the State Secretariat for Justice and Citizenship of Roraima and by the prison unit’s director.

## RESULTS

All 179 women deprived of liberty from the Boa Vista’s Public Female Prison were invited to participate in the study, and a total of 168 (93.8%) agreed to participate. The women’s age ranged from 18 to 60 years, with a mean of 36.5 (±9.5) years and a median of 31 years. Half the sample was single, 46.4% were up to 30 years of age, 37.5% studied until elementary school, and 44.6% were evangelical. A minority (2.4%) declared themselves as transgender. Regarding the nature of conviction, 54.2% (n = 91) of the participants were sentenced, and the average sentence time was 9.6 years. The other inmates (45.8%) were in provisional custody (without conviction). The mean incarceration length (n = 163) up to the time of the study was 2.7 years. Table 1 describes the sociodemographic and judicial characteristics.

**Table 1:** Description of the sociodemographic and criminal characteristics of women deprived of liberty of the Public Women’s Jail of Boa Vista, Roraima, Brazil, 2017.

Sample characteristics (n = 168)	n	%
Cisgender women	164	97.6
Transgender women	4	2.4
Age Up to 30 years Over 30 years	78 90	46.4 53.6
Place of birth Roraima Other states in Brazil Other countries	82 81 5	48.8 48.2 3.0
Marital status Married/common-law marriage Single Steady relationship Separated/widowed No information available	48 84 13 4 19	28.6 50.0 7.7 2.4 11.3
Schooling level Did not study — illiterate Some elementary school (up to 5 years of schooling) Elementary school High School or some high school College or some college	8 20 43 79 18	4.8 11.9 25.6 47.0 10.7
Religion Evangelical Catholic Others No religion/no wish to inform	75 50 3 40	44.6 29.8 1.8 23.8
Current sentencing time (years) (n = 82) Up to 2 > 2 to 4 > 4 to 6 > 6 to 10 > 10	1 4 18 26 33	1.2 5.0 22.5 32.2 40.2
Current incarceration time (years) (n = 163) Up to 2 > 2 to 4 > 4 to 6 > 6 to 10	122 31 3 7	74.8 19.0 1.8 4.2

The seroprevalence of some STI in the sample was 20.2% (n = 34). The prevalence of HIV infection was 4.7% (n = 8); syphilis, 15.5% (n = 26); and hepatitis B, 0.0% ( Table 2 ). The prevalence of HIV and syphilis co-infection was 1.2% (n = 2). Among the HIV-positive, 75% (n = 6) knew the diagnosis before incarceration.

**Table 2:** Prevalence of HIV, syphilis and hepatitis B in the Public Women’s Jail of Boa Vista, Roraima, Brazil, 2017.

Prevalence (n = 168)	n	%	95%CI
Some STI (combined)	34	20.2	12.52–28.73
HIV	8	4.7	2.08–9.17
Syphilis	26	15.5	10.37–21.85
Hepatitis B	0	0.0	-

STI: sexually transmitted infection

The univariate analysis concerned the sociodemographic characteristics, knowledge and perception about HIV/AIDS, syphilis and hepatitis B, and considered the seroprevalence of some of these STI as the main outcome ( Table 3 ). Among the sociodemographic characteristics, two variables stood out: being over 30 years of age almost tripled the chance of having an STI (26.6% – 10.3%, respectively; p = 0.007); and low schooling (up to elementary school) also significantly increased the prevalence of STI compared with women with high school or higher education (29.6% – 11.3%, respectively; p = 0.003). Lacking a steady partner had no influence on this outcome.

**Table 3:** Univariate analysis among sociodemographic characteristics, knowledge, perception and risk behavior, in relation to the seroprevalence of STI in women deprived of liberty of the Public Women’s Jail of Boa Vista, Roraima, Brazil, 2017.

	n (%)	Prevalence of STI (%)	p	OR	95%CI
Sociodemographic characteristics (n = 168)					
Over 30 years Less than 30 years	99 (58.9) 69 (41.0)	26.6 10.3	0.007	3.16	1.35–7.96
Schooling up to elementary school High school/College	71 (42.2) 97 (57.8)	29.6 11.3	0.003	3.25	1.44 –7.55
Has a steady relationship Does not have	80 (46.7) 84 (52.4)	19.6 18.0	NS	1.10	0.49–2.56
Knowledge about HIV/AIDS, syphilis and hepatitis B (n = 168)					
Knows what AIDS is Does not know Knows	32 (19.0) 136 (81.0)	34.4 15.4	0.01	2.84 1	1.16–6.79
Knowledge about condom use (% of hits) Up to 50% More than 50%	93 (55.3) 75 (44.7)	34.6 16.3	0.02	3.65 1	1.16–7.70
Does not know that condom prevents the transmission of HIV/AIDS Syphilis Hepatitis B	39 (23.2) 56 (33.3) 83 (44.8)	25.6 25.0 21.7	NS NS NS	1.67 1.73 1.40	0.68–3.90 0.77–3.83 0.64–3.09
The person with AIDS does not always present the symptoms of the disease Does not know Knows	103 (61.3) 65 (38.7)	20.2 17.2	NS	1.21 1	0.54–2.81
HIV cannot be transmitted by soaps, towels and toiletries Does not know Knows	56 (33.3) 112 (66.6)	23.6 16.1	NS	1.61 1	0.70–3.60
Sharing syringe/needle by several people can transmit AIDS Does not know Knows	6 (3.5) 162 (96.5)	16.7 19.1	NS	0.84 1	0.03–6.38
AIDS is a disease that has treatment Does not know Knows	30 (17.8) 138 (82.2)	16.7 19.6	NS	0.82 1	0.25–2.26
AIDS is a disease that has no cure Does not know Knows	29 (17.2) 139 (82.8)	24.2 18.0	NS	1.44 1	0.52–3.70
Knows what AIDS is Does not know Knows	67 (39.8) 101 (60.2)	20.9 17.8	NS	1.21 1	0.54–2.66
Knows what hepatitis B is Does not know Knows	87 (51.7) 81 (48.3)	23.0 14.8	NS	1.71 1	0.77–3.87
Knowledge about HIV/AIDS, syphilis and hepatitis B (n = 168)					
Thinks is not at risk of getting the following disease HIV Syphilis Hepatitis B	81 (48.2) 91 (54.1) 95 (56.5)	20.0 26.4 24.5	NS 0.01 0.04	1.19 2.98 1.98	0.54–2.66 1.27–7.51 1.11–3.95
AIDS is a disease of gays, prostitutes and drug addicts Agrees Does not agree	22 (13.9) 146 (86.9)	22.7 18.5	NS	1.29 1	0.39–3.71
Is afraid to get AIDS No Yes	23 (13.6) 145 (86.4)	30.4 17.2	NS	2.09	0.73–5.57
Older people do not have to worry about AIDS Agrees Does not agree	21 (12.5) 147 (87.5)	25.0 18.4	NS	1.47 1	0.44–4.32
Thinks AIDS is a serious disease No Yes	24 (14.2) 144 (85.8)	21.7 18.7	NS	1.20 1	0.36–3.42
Risky behavior (n = 168)					
Makes use of injectable cocaine No	5 (2.9) 163 (87.1)	40.0 18.4	0.02	2.95 1	1.04–18.4
Suffered sexual abuse in prison No	4 (8.3) 163 (91.7)	50.0 18.4	0.02	4.47 1	1.16 -32.7
Receives intimate visit Does not receive	19 (11.3) 149 (88.7)	26.3 18.1	NS	1.60	0.42–5.26
Uses condom Always Not always	59 (39.6) 90 (60.4)	27.1 14.7	NS	0.46 1	0.21–1.01
Incorrect condom use (starts using it after penetration) Correct condom use	35 (20.8) 133 (79.2)	22.5 16.5	NS	1.47 1	0.67–3.19
Woman who has sex with woman Woman who has sex with man	64 (39.3) 102 (60.7)	14.0 22.3	NS	0.56 1	0.24–1.32
Sexual intercourse inside the prison No	4 (2.3) 163 (97.7)	18.9 19.0	NS	0.99 1	0.44–2.23

NS: not significant

Regarding knowledge about STI, lacking knowledge about AIDS more than doubled the chance of infection (OR = 2.84; 95%CI 1.16–6.79) compared with those who knew the disease (prevalence of STI: 34.4% *versus* 15.4%, respectively; p = 0.01). Lacking knowledge about condom use was also associated with a higher chance of having STI: presenting less than 50% of correct answers in questions of knowledge about condom use significantly increased the chance of having STI compared with those with a better performance (34.6% *versus* 16.3%; respectively; p = 0.02; OR = 3.65; 95%CI 1.16–7.97).

Distortions of perception about STI were not associated with infection. However, believing that there is no risk of contracting syphilis almost tripled the chance of positive STI tests (OR = 2.98; 95%CI 1.27–7.51), and believing that there is no risk of contracting hepatitis B also significantly increased the chance of infection (26.4% *versus* 10.3%, respectively; p = 0.04). Regarding risk behavior, inmates who reported using injectable cocaine had a significantly higher prevalence of STI than those who did not (40.0% *versus* 18.4%, respectively; p = 0.02), and were almost three times more likely to test positive for STI (OR = 2.95; 95%CI 1.04–18.4). The variable with the highest correlation with a positive STI test was the report of sexual abuse in prison. Inmates who reported being abused were four times more likely to be infected (OR = 4.47; 95%CI 1.06–32.7) and showed a 50% (p = 0.02) prevalence of STI.

The explanatory variables that showed a significant correlation with the prevalence of STI in univariate analysis were reanalyzed in a multivariate analysis ( Table 4 ). Independent associated factors confirmed for positive results in STI tests were: being over 30 years of age (adjusted OR: 2.57; 95%CI 1.03–6.40); schooling up to elementary school (adjusted OR: 2.77; 95%CI 1.08–5.05); little knowledge about condom use (adjusted OR: 2.37; 95%CI 1.01–7.31); and believe that there is no risk of contracting syphilis (adjusted OR: 2.36; 95%CI 1.08–6.50).

**Table 4:** Univariate analysis among sociodemographic characteristics, knowledge, perception and risky behavior, in relation to the seroprevalence of STI in women deprived of liberty of the Public Women’s Jail of Boa Vista, Roraima, Brazil, 2017.

Explanatory variable	Adjusted OR	95%CI	p*
Over 30 years	2.57	1.03–6.40	0.04
Schooling up to elementary school	2.77	1.08–5.05	0.03
Little knowledge about condom use	2.37	1.01–7.31	0.04
Thinks has no risk of getting syphilis.	2.36	1.08–6.50	0.03
Sexual abuse	4.93	0.48–49.8	NS
Does not know about HIV	1.88	0.71–5.08	NS
Cocaine use	2.72	0.31–23.4	NS
Thinks has no risk of getting hepatitis B	0.87	0.67–1.45	NS

* Statistical significance level was set at 0.05%.

NS: not significant

## DISCUSSION

Brazil has the third largest prison population in the world – in absolute terms, second only to the United States and China ^11^ –, and understand the epidemiology of the main diseases of this population, especially of infectious diseases, gains notorious relevance in public health. This was the first study to analyze the epidemiology of HIV/STI in inmates in Roraima, highlighting its high participation rate (93.8% of the female population deprived of liberty). Our sample revealed a high prevalence of STI. One in five women serving time in the Public Female Prison in Boa Vista had a positive result in the rapid test of some STI – higher prevalence than that reported in a similar study conducted in Ceará in 2010, with 155 inmates, in which 13.5% of the women had some STI ^12^ . To understand the magnitude of the prevalence of STI in persons deprived of liberty, it is worth comparing with the results of a study conducted with the general population of Roraima. Fonseca et al. ^13^ conducted a household survey of STI seroprevalence in Roraima in 2017. Of the 727 adults surveyed, 5.8% presented some STI, a percentage significantly lower than in the population deprived of liberty of the same state. Considering only the women included in this study (n = 420), the prevalence of STI was 5.5%.

Regarding HIV, the prevalence of infection in prison (4.7%) was five times higher than in the general population of Roraima (0.9%) ^13^ . Although studies with prison populations are scarce, in the 1980s and 1990s some reported high prevalence of HIV infection in female prisons in Brazil – in São Paulo, for example, Queiroz et al. ^14^ found a rate of 18.3% in 1987. To our knowledge, the highest prevalence of HIV infection ever described in Brazilian female prisons was 26%, also in São Paulo, in 1997 ^5^ . More recent studies suggest a trend of reduced prevalence of HIV infection in female prisons in recent decades in Brazil. In 2012, in Mato Grosso do Sul, the prevalence of this infection was only 0.7% ^15^ , and in a 2014 study conducted in 12 prisons in cities in the Midwest region, it reached 1.9% ^16^ . Neves et al. ^17^ described 4.9% of HIV positivity in a female prison in Goiás, a data similar to that found in Roraima. On the other hand, a nationwide study on the profile of the Brazilian female penitentiary population in 15 prisons (from nine states), conducted in 2015 and 2016, reported a prevalence of 2.3% ^18^ .

The prevalence of syphilis found in the present study was almost five times higher than that of the general population of Roraima (3.2%) ^13^ and almost three times higher than the national prevalence estimates (5.2%) ^19^ . Although very high, this rate is still lower than that of women deprived of liberty in Recife (PE), in 2013 (23.8%) ^20^ , and in São Paulo, in 2000 (22.8%) ^7^ . However, it is similar to the prevalence found by Miranda et al. ^21^ in Espírito Santo, which was 15.5% in 1997. The absence of a hepatitis B virus (HBV) infection marker in the studied population drew much attention, as HBV infection has transmission routes similar to those of HIV, with co-infection being common. Besides, the evidence indicates a high prevalence of HBV in the population deprived of liberty, as in prisons in Goiânia (GO) (26.4%) and Ribeirão Preto (SP) (19.5%) ^8 , 22^ . A significant proportion of the women studied believe that they are not at risk of contracting hepatitis B, to the point where the chance of infection is statistically significant – which can therefore be considered a future risk of HBV infection. It is worth mentioning that we lack knowledge of these women’s vaccination status.

Most of the participants in the present study had a low educational level, corroborating evidence from the literature. In Freire’s ^18^ study, 61.4% of the population of 15 female prisons in nine Brazilian states had not accessed secondary education. Recent national data show that 65% of the Brazilian female prison population has not yet accessed high school, having completed, at most, primary education ^10^ . Low schooling was one of the variables with the highest association with STI in our sample, tripling the chance of infection. Camargo et al. ^23^ reported that most HIV-positive inmates have incomplete first grade. The low level of education hinders access to and understanding information on STI, limiting the adoption of preventive behavior ^24^ . Early school dropout due to involvement in illicit activities can contribute to this phenomenon in the female population deprived of liberty. As such, a context of impoverishment of the AIDS epidemic emphasizes the vulnerability of this group ^15^ .

In this sense, one third of the inmates who reported not knowing about AIDS tested positive for some STI, and this lack of knowledge almost tripled the prevalence of infection. Kuznetzova et al. ^25^ , when analyzing the inmates’ knowledge about HIV/AIDS, described numerous misconceptions that fueled false beliefs about the forms of prevention and transmission of the disease. Behavioral studies confirm that attitude is modulated by several factors, such as beliefs, emotions and values; but the influence of knowledge on behavior is fundamental, positively or negatively ^26^ .

Regarding the perception of STI, although distortions were frequent, they did not correlate strongly with the chance of infection in this study, except for self-perception of invulnerability. The feeling of not being at risk of contracting syphilis and hepatitis B more than doubled the chance of infection. Thus, the correlations between ignorance, distorted perceptions and STI positivity evidenced in our study validate Pelto and Pelto’s observations ^27^: causal or mistaken attitudes can be considered a product of beliefs or ignorance. The same can be interpreted regarding knowledge deficit about condom use. Approximately 20% of the sample were unaware that using condoms prevents HIV, syphilis and hepatitis B transmission. The prevalence of infection was significantly higher in women with knowledge deficits about the main form of preventing STI. The World Health Organization (WHO) recommends, in the guide on HIV/AIDS infection in prison, that both inmates and professionals working in the prison system should receive continued education on HIV prevention and transmission ^28^ . In most prisons in Brazil, health education actions aimed at controlling STI are rare. The reality of health in prisons is still precarious and neglected ^29^ .

As expected, the use of injectable drugs was correlated with STI in our sample, suggesting that needle sharing is an important vector in this group, either before or during prison. There is evidence that exposure to risk behaviors by individuals deprived of liberty usually begins when they are free individuals, before incarceration ^15^ . However, one variable stood out, both for having a stronger correlation with the chance of having STI and for demonstrating the vulnerability of these women during their imprisonment: 8.3% of them reported having been sexually abused in prison, and half of them tested positive for STI, which represents an almost five times greater chance of infection compared with those without reported abuse. Overcrowding, exposure to physical violence, poor lighting, restricted access to justice and medical services contribute to the increased vulnerability of this population to STI, such as the high risk of sexual abuse within prison walls ^30^ . This situation reflects a serious public health issue, since the prison system can function as an environment conducive to the spread of STI, as a place of agglomeration of these infections and, finally, as a disseminating focus for the general population ^31^ .

As a limitation of this study, we point out the evaluation of STI restricted to HIV, syphilis and hepatitis B, disregarding the remaining infections. Behavioral aspects, such as alcohol consumption, were also unaddressed. Despite the researchers’ best efforts to obtain valid information there may have been response bias, as the study addressed sensitive issues related to risk behavior.

The population deprived of liberty is a group of high vulnerability to STI. The high prevalence of these infections can be explained by knowledge deficits on the subject, distorted perceptions and conditions peculiar to imprisonment, which result in risky behavior. We emphasize the need to implement educational programs for preventing, diagnosing and treating STI for this population. We consider important to expand the study to other infections, as well as a similar study for the male population deprived of liberty in the state of Roraima.
