# Supplementary material for: Sexually transmitted infections in women deprived of liberty in Roraima, Brazil
Source: Rev Saude Publica. 2020 Oct 28;54:105. doi: 10.11606/s1518-8787.2020054002207 (PMC7593044; doi:10.11606/s1518-8787.2020054002207)
Supplement: Translation

# Translation: Infecções sexualmente transmissíveis em mulheres privadas de liberdade em Roraima

**Authors:** Maria Soledade Garcia Benedetti, Audrey Stella Akemi Nogami, Beatriz Belo da Costa, Herbert Iago Feitosa da Fonsêca, Igor dos Santos Costa, Itallo de Souza Almeida, Luana de Miranda, Matheus Mychael Mazzaro Conchy, Renan da Silva Bentes, Suzani Naomi Higa, Tháles de Souza Israel, Allex Jardim da Fonseca

**Affiliations:** I Brasil Universidade Federal de Roraima. Centro de Ciências da Saúde. Curso de Medicina. Boa Vista, RR, Brasil

**Keywords:** Mulheres, Prisões, Doenças Sexualmente Transmissíveis, epidemiologia, Fatores de Risco, Disparidades nos Níveis de Saúde

## Abstract

**OBJETIVO:**

Avaliar a prevalência de infecção por vírus da imunodeficiência humana (HIV), sífilis e hepatite B entre mulheres privadas de liberdade do estado de Roraima e sua correlação com percepções, conhecimento e fatores comportamentais.

**MÉTODO:**

Trata-se de estudo de corte transversal, com amostragem sistemática simples, realizado na Cadeia Pública Feminina de Boa Vista, estado de Roraima, no ano de 2017. Foram avaliadas 168 detentas (93,8% da população) por meio de entrevista face a face e testes rápidos.

**RESULTADOS:**

A prevalência de alguma infecção sexualmente transmissível (IST) foi de 20,2%, sendo 4,7% de HIV, 15,5% de sífilis, e 0,0% de hepatite B. A análise multivariada confirmou como fatores de risco para adquirir uma IST: ter mais de 30 anos de idade [ *odds ratio* (OR) ajustada: 2,57; IC95% 1,03–6,40); baixa escolaridade (OR ajustada: 2,77; IC95% 1,08–5,05); pouco conhecimento sobre o uso da camisinha (OR ajustada: 2,37; IC95% 1,01–7,31); e achar que não há risco de contrair sífilis (OR ajustada: 2,36; IC95% 1,08–6,50).

**CONCLUSÃO:**

A população privada de liberdade constitui um grupo de alta vulnerabilidade às IST. A elevada prevalência dessas infecções pode ser explicada por déficits de conhecimento sobre o assunto, percepções distorcidas e condições peculiares ao aprisionamento, que resultam em comportamento de risco. Ressalta-se a necessidade de implantar programas educativos de prevenção, diagnóstico e tratamento de IST para essa população.

## INTRODUÇÃO

O aparecimento da síndrome da imunodeficiência adquirida (aids) nos presídios ocorreu concomitantemente ao seu surgimento na comunidade ^1^ . Os primeiros casos de aids em presídios foram registrados no início dos anos 1980 nos Estados Unidos, nas cidades de Nova York e Nova Jersey ^2^ . Desde então, diversos estudos têm alertado que a prevalência de HIV e outras infecções sexualmente transmissíveis (IST) entre presos condenados é significativamente maior que na população em geral, podendo contribuir para isso comportamentos de risco anteriores e durante o cárcere ^3^ .

No Brasil, estudos pontuais realizados em unidades prisionais femininas de algumas regiões revelaram prevalência elevada de IST em mulheres encarceradas: de 0,0% a 26% para HIV ^4 , 5^ , 2,2% a 22,8% para sífilis ^6 , 7^ , e 3,8% a 26,4% para hepatite B ^4 , 8^ . Entretanto, não estão claros na literatura médica os fatores associados a maior risco de contrair HIV ou IST nessa população em especial, sobretudo nas unidades prisionais femininas, onde o risco de transmissão de IST entre as detentas é significativamente menor em comparação aos detentos masculinos. Adicionalmente, não se pode extrapolar o conhecimento de fatores associados à transmissão de IST na população geral para a população carcerária em virtude da condição peculiar de privação de liberdade. O encarceramento pode influenciar de modo multifatorial a vulnerabilidade dessas mulheres, não somente por terem menor acesso ao conhecimento sobre mecanismos de prevenção e transmissão, mas também por exposição a violência sexual, distorção de percepções de risco ou simplesmente acesso restrito a preservativos e consultas com profissionais de saúde.

Estudos sobre a epidemiologia de HIV/IST em populações prisionais são ainda escassos, em especial na região Norte do Brasil. Levando em consideração as evidências alarmantes da vulnerabilidade das mulheres encarceradas, o estabelecimento prisional pode ser considerado um local de especial interesse científico, além de oportuno para diagnóstico e tratamento das infecções. O objetivo deste estudo é avaliar a prevalência de infecção por HIV, sífilis e hepatite B em mulheres privadas de liberdade no estado de Roraima e sua correlação com conhecimento, percepção, comportamento e fatores sociodemográficos.

## MÉTODOS

### Desenho do Estudo

Trata-se de estudo de corte transversal desenhado para avaliar a soroprevalência de infecção por HIV/IST entre mulheres privadas de liberdade no estado de Roraima, por meio de testes rápidos e questionário abordando os fatores associados à infecção, realizado em 2017.

### População

Roraima é um estado do extremo norte da Amazônia Legal brasileira e tem uma população de cerca de 500 mil habitantes, conforme estimativa do Instituto Brasileiro de Geografia e Estatística (IBGE) em 2017. Destaca-se por ter duas fronteiras internacionais (com a Venezuela e a Guiana) e por abrigar a maior população indígena aldeada do Brasil (aproximadamente 15% da população do estado).

A pesquisa foi realizada na Cadeia Pública Feminina de Boa Vista (capital), que é o único estabelecimento penal feminino do estado e abrigava 179 detentas à época do estudo. Roraima ocupa o penúltimo lugar no *ranking* nacional do número de mulheres privadas de liberdade, abrigando 0,4% da população feminina encarcerada no país; entretanto, tem a quinta maior taxa de aprisionamento do Brasil, de 67 para cada 100 mil mulheres. A taxa de ocupação do sistema prisional feminino do estado é de 228%, quase o dobro da média nacional para unidades femininas. As mulheres representam 7,2% da população prisional do estado, e quase metade delas (48%) está em condenação; as demais estão sentenciadas ao regime fechado (13%), ao regime semiaberto (12%) e ao regime aberto (28%) ^9^ .

A pesquisa ocorreu nos meses de outubro e novembro de 2017, antes, portanto, da migração maciça de refugiados venezuelanos para o Brasil através de Roraima.

### Amostra e Amostragem

Para calcular o tamanho da amostra, a prevalência de IST na população-alvo foi estimada em 20%, com base em estudos semelhantes. Considerando intervalo de confiança de 95% e erro aceitável de 5%, obteve-se o tamanho amostral mínimo de 254 detentas. Ajustando a meta amostral para uma população pequena (n = 179), obteve-se um tamanho de amostra mínimo de 146 participantes. Considerando perdas de amostra e rejeição de participação nos testes de até 10%, a meta amostral final foi de 161 mulheres. O método de amostragem foi sistemático simples: todas as detentas de todas as celas foram convidadas a participar, sem seleção e de forma consecutiva, conforme disposição das celas nos blocos da unidade prisional.

### Procedimento de Pesquisa

Todas as detentas foram individualmente convidadas a participar do estudo, sob escolta de agentes penitenciários, em suas celas. Em caso de aceitação, a detenta era transferida para sala reservada onde, após amplo esclarecimento, registrava seu consentimento formalmente. Cada participante respondeu a um questionário não identificado nominalmente, com privacidade, sendo a primeira parte deste aplicada pelo pesquisador (entrevista face a face), abordando dados demográficos, conhecimento e percepção sobre HIV/IST. A segunda parte do questionário era autoaplicável, abordando questões de exposição sexual e uso de drogas, e deveria ser depositada sigilosamente em urna lacrada. Nesta etapa, as participantes analfabetas foram auxiliadas pelos médicos pesquisadores. O questionário semiestruturado foi adaptado do estudo de Miranda et al. ^10^ .

Posteriormente, as participantes foram convidadas a realizar o teste rápido de HIV, que utiliza a tecnologia imunocromatográfica, por meio de ensaio qualitativo, para detecção de anticorpos específicos de HIV-1/2 (TR DPP ^®^ HIV-1/2, Bio-Manguinhos, Rio de Janeiro, Brasil); de sífilis (Alere Sífilis), por meio da detecção qualitativa de anticorpos IgG, IgM e IgA contra o *Treponema pallidum* , caracterizado como um teste treponêmico (Importador Alere S/A, São Paulo, Brasil); e de hepatite B (Vikia ^®^ HBsAg, BioMérieux SA, França), por meio da detecção qualitativa do antígeno HBs.

Foi realizada punção digital com lanceta estéril, além de aconselhamento pré e pós-teste, e o resultado foi informado em até 30 minutos, de forma individualizada. Os resultados não reagentes foram considerados negativos. Já nos casos de resultado reagente ou inconclusivo para HIV, houve retestagem pelo teste rápido ou imunocromatográfico Bioeasy para detecção qualitativa de anticorpos de todos os isótopos (IgG, IgM e IgA) específicos para HIV-1, incluindo o subtipo O, e HIV-2, simultaneamente. Na positividade deste teste, o resultado foi considerado confirmado como HIV. Para hepatite B, o teste rápido positivo é confirmatório e, para sífilis, o teste rápido é de triagem sorológica. Todos os casos positivos foram encaminhados para serviço especializado.

### Análise de Dados

Os dados de cada participante foram armazenados e analisados no software Epi Info ^TM^ , versão 7.2.2.6. Foi calculada a prevalência de HIV, sífilis e hepatite B na amostra, e realizou-se análise descritiva das características sociodemográficas, da natureza da prisão, do tipo de regime, do tempo de condenação e do tempo de encarceramento (em meses), sendo utilizadas medidas de tendência central para variáveis contínuas, e percentuais para variáveis categóricas.

O teste qui-quadrado foi utilizado para comparar diferenças de proporções de variáveis categóricas, e foi considerado nível de significância de 5%. *Odds ratio* (OR) e intervalo de confiança de 95% (IC95%) foram calculados em análise univariada, sendo a OR ajustada em análise multivariada por regressão logística. O critério de seleção de variáveis explicativas para entrada na análise multivariada foi o valor crítico de p < 0,15 na análise univariada.

### Aspectos Éticos

O estudo foi aprovado pelo Comitê de Ética em Pesquisa envolvendo seres humanos da Universidade Federal de Roraima (UFRR) – Parecer nº 1.797.708 e CAAE 44535315.3.0000.5302. Foi também autorizado formalmente pela Secretaria de Estado da Justiça e da Cidadania de Roraima e pela direção da unidade prisional.

## RESULTADOS

Foram convidadas a participar da pesquisa todas as 179 mulheres privadas de liberdade da Cadeia Pública Feminina de Boa Vista, e um total de 168 (93,8%) aceitou participar do estudo. A idade das mulheres variou de 18 a 60 anos, com média de 36,5 (±9,5) anos e mediana de 31 anos. Metade da amostra era solteira, 46,4% tinham até 30 anos de idade, 37,5% estudaram até o ensino fundamental, e 44,6% eram evangélicas. Uma minoria (2,4%) se autodeclarou transgênero. Com relação à natureza da prisão, 54,2% (n = 91) das participantes eram sentenciadas, e o tempo médio de condenação foi de 9,6 anos. As demais detentas (45,8%) estavam em prisão provisória (sem condenação). O tempo médio de encarceramento (n = 163) até o momento da pesquisa era de 2,7 anos. A Tabela 1 apresenta a descrição das características sociodemográficas e penais.

**Tabela 1:** Descrição das características sociodemográficas e penais das mulheres privadas de liberdade da Cadeia Pública Feminina de Boa Vista, Roraima, Brasil, 2017.

Características da amostra (n = 168)	n	%
Mulheres cisgênero	164	97,6
Mulheres transgênero	4	2,4
Idade Até 30 anos Mais de 30 anos	78 90	46,4 53,6
Naturalidade Roraima Outros estados do Brasil Outros países	82 81 5	48,8 48,2 3,0
Estado marital Casada/união estável Solteira Namoro fixo Separada/viúva Sem informação	48 84 13 4 19	28,6 50,0 7,7 2,4 11,3
Escolaridade Não estudou - analfabeta Ensino fundamental incompleto (até 5 anos de estudo) Ensino fundamental completo/semi-completo Ensino médio (completo/incompleto) Ensino superior (completo/incompleto)	8 20 43 79 18	4,8 11,9 25,6 47,0 10,7
Religião Evangélica Católica Outras Sem religião/não deseja informar	75 50 3 40	44,6 29,8 1,8 23,8
Tempo de condenação atual (anos) (n = 82) Até 2 > 2 a 4 > 4 a 6 > 6 a 10 > 10	1 4 18 26 33	1,2 5,0 22,5 32,2 40,2
Tempo de encarceramento atual (anos) (n = 163) Até 2 > 2 a 4 > 4 a 6 > 6 a 10	122 31 3 7	74,8 19,0 1,8 4,2

A soroprevalência de alguma IST na amostra foi de 20,2% (n = 34). A prevalência de infecção por HIV foi de 4,7% (n = 8); de sífilis, de 15,5% (n = 26); e de hepatite B, 0,0% ( Tabela 2 ). A prevalência de coinfecção de HIV e sífilis foi de 1,2% (n = 2). Entre as portadoras de HIV, 75% (n = 6) conheciam o diagnóstico antes do encarceramento.

**Tabela 2:** Prevalência de HIV, sífilis e hepatite B na Cadeia Pública Feminina de Boa Vista, Roraima, Brasil, 2017.

Prevalência (n = 168)	n	%	IC95%
Alguma IST (combinadas)	34	20,2	12,52–28,73
HIV	8	4,7	2,08–9,17
Sífilis	26	15,5	10,37–21,85
Hepatite B	0	0,0	-

IST: infecção sexualmente transmissível

A análise univariada foi realizada entre características sociodemográficas, conhecimento e percepção sobre HIV/aids, sífilis e hepatite B, e considerou a soroprevalência de alguma dessas IST como desfecho principal ( Tabela 3 ). Dentre as características sociodemográficas, duas variáveis se destacaram: ter mais de 30 anos de idade quase triplicou a chance de ter uma IST (26,6% *versus* 10,3%, respectivamente; p = 0,007); e a baixa escolaridade (até ensino fundamental) também aumentou significativamente a prevalência de IST em relação às mulheres com ensino médio ou superior (29,6% *versus* 11,3%, respectivamente; p = 0,003). Não ter parceiro fixo não influenciou esse desfecho.

**Tabela 3:** Análise univariada entre as características sociodemográficas, conhecimento, percepção e comportamento de risco, em relação à soroprevalência de IST em mulheres privadas de liberdade da Cadeia Pública Feminina de Boa Vista, Roraima, Brasil, 2017.

	n (%)	Prevalência (%) de IST	p	OR	IC95%
Características sociodemográficas (n = 168)					
Mais de 30 anos de idade Menos de 30 anos	99 (58,9) 69 (41,0)	26,6 10,3	0,007	3,16	1,35–7,96
Escolaridade até ensino fundamental Ensino médio/superior	71 (42,2) 97 (57,8)	29,6 11,3	0,003	3,25	1,44 –7,55
Possui parceiro(a) fixo Não possui	80 (46,7) 84 (52,4)	19,6 18,0	NS	1,10	0,49–2,56
Conhecimento sobre HIV/aids, sífilis e hepatite B (n = 168)					
Saber o que é aids Não sabe Sabe	32 (19,0) 136 (81,0)	34,4 15,4	0,01	2,84 1	1,16–6,79
Conhecimentos sobre o uso da camisinha (% de acertos) Até 50% Mais de 50%	93 (55,3) 75 (44,7)	34,6 16,3	0,02	3,65 1	1,16–7,70
Não saber que o uso da camisinha impede a transmissão de HIV/ aids Sífilis Hepatite B	39 (23,2) 56 (33,3) 83 (44,8)	25,6 25,0 21,7	NS NS NS	1,67 1,73 1,40	0,68–3,90 0,77–3,83 0,64–3,09
A pessoa com aids nem sempre apresenta os sintomas da doença Não sabe Sabe	103 (61,3) 65 (38,7)	20,2 17,2	NS	1,21 1	0,54–2,81
HIV não pode ser transmitido por sabonetes, toalhas e sanitários Não sabe Sabe	56 (33,3) 112 (66,6)	23,6 16,1	NS	1,61 1	0,70–3,60
Compartilhar seringa/agulha por diversas pessoas pode transmitir aids Não sabe Sabe	6 (3,5) 162 (96,5)	16,7 19,1	NS	0,84 1	0,03–6,38
A aids é uma doença que tem tratamento Não sabe Sabe	30 (17,8) 138 (82,2)	16,7 19,6	NS	0,82 1	0,25–2,26
A aids é uma doença que não tem cura Não sabe Sabe	29 (17,2) 139 (82,8)	24,2 18,0	NS	1,44 1	0,52–3,70
Saber o que é sífilis Não sabe Sabe	67 (39,8) 101 (60,2)	20,9 17,8	NS	1,21 1	0,54–2,66
Saber o que é hepatite B Não sabe Sabe	87 (51,7) 81 (48,3)	23,0 14,8	NS	1,71 1	0,77–3,87
Percepção sobre HIV/aids, sífilis e hepatite B (n = 168)					
Achar que não tem risco tem risco de pegar a seguinte doença HIV Sífilis Hepatite B	81 (48,2) 91 (54,1) 95 (56,5)	20,0 26,4 24,5	NS 0,01 0,04	1,19 2,98 1,98	0.54–2,66 1,27–7,51 1,11–3,95
A aids somente pega em gays, prostitutas e viciados em drogas Concorda Não concorda	22 (13,9) 146 (86,9)	22,7 18,5	NS	1,29 1	0,39–3,71
Ter medo de pegar aids Não Sim	23 (13,6) 145 (86,4)	30,4 17,2	NS	2,09	0,73–5,57
As pessoas mais velhas não precisam se preocupar com a AIDS Concorda Não concorda	21 (12,5) 147 (87,5)	25,0 18,4	NS	1,47 1	0,44–4,32
Achar que a aids é uma doença grave Não Sim	24 (14,2) 144 (85,8)	21,7 18,7	NS	1,20 1	0,36–3,42
Comportamento de risco (n=168)					
Faz uso de cocaína injetável Não	5 (2,9) 163 (87,1)	40,0 18,4	0,02	2,95 1	1,04–18,4
Sofreu abuso sexual na prisão Não	4 (8,3) 163 (91,7)	50,0 18,4	0,02	4,47 1	1,16 -32,7
Recebe visita íntima Não recebe	19 (11,3) 149 (88,7)	26,3 18,1	NS	1,60	0,42–5,26
Usa camisinha Sempre Nem sempre	59 (39,6) 90 (60,4)	27,1 14,7	NS	0,46 1	0,21–1,01
Uso incorreto da camisinha (iniciar uso após a penetração) Uso correto da camisinha	35 (20,8) 133 (79,2)	22,5 16,5	NS	1,47 1	0,67–3,19
Mulher que faz sexo com mulher Mulher que faz sexo com homem	64 (39,3) 102 (60,7)	14,0 22,3	NS	0,56 1	0,24–1,32
Relação sexual dentro do presídio Não	4 (2,3) 163 (97,7)	18,9 19,0	NS	0,99 1	0,44–2,23

NS: não significativo

Com relação ao conhecimento sobre IST, não saber o que é aids mais que dobrou a chance de infecção (OR = 2,84; IC95% 1,16–6,79) em comparação com as que conheciam a doença (prevalência de IST: 34,4% *versus* 15,4%, respectivamente; p = 0,01). O déficit de conhecimento sobre o uso de camisinha também se associou a maior chance de ter IST: apresentar menos de 50% de acertos nas questões de conhecimento sobre o uso de preservativo aumentou significativamente a chance de ter IST em comparação àquelas participantes com melhor desempenho (34,6% *versus* 16,3%; respectivamente; p = 0,02; OR = 3,65; IC95% 1,16–7,97).

As distorções de percepção sobre IST não apresentaram associação com a infecção. Entretanto, acreditar que não há risco de contrair sífilis quase triplicou a chance de positividade nos testes de IST (OR = 2,98; IC95% 1,27–7,51), e acreditar que não há risco de contrair hepatite B também aumentou significativamente a chance de infecção (26,4% *versus* 10,3%, respectivamente; p = 0,04). Quanto ao comportamento de risco, as detentas que referiram fazer uso de cocaína injetável apresentaram prevalência de IST significativamente maior do que as que não o fazem (40,0% *versus* 18,4%, respectivamente; p = 0,02), e esse primeiro grupo apresentou quase três vezes mais chance de positividade nos testes de IST (OR = 2,95; IC95% 1,04–18,4). A variável com maior correlação com teste positivo de IST foi o relato de abuso sexual na prisão. As detentas que relataram sofrer abuso apresentaram chance quatro vezes maior de infecção (OR = 4,47; IC95% 1,06–32,7) e elevada prevalência de IST de 50% (p = 0,02).

As variáveis explicativas que apresentaram correlação significativa com a prevalência de IST em análise univariada foram reanalisadas em análise multivariada ( Tabela 4 ). Confirmaram-se como fatores associados independentes para resultado positivo nos testes de IST: ter mais de 30 anos de idade (OR ajustada: 2,57; IC95% 1,03–6,40); escolaridade até o ensino fundamental (OR ajustada: 2,77; IC95% 1,08–5,05); pouco conhecimento sobre o uso da camisinha (OR ajustada: 2,37; IC95% 1,01–7,31); e achar que não há risco de contrair sífilis (OR ajustada: 2,36; IC95% 1,08–6,50).

**Tabela 4:** Análise multivariada entre as características sociodemográficas, conhecimento e percepção sobre HIV/AIDS, sífilis e hepatite B, e comportamento de risco considerando a soroprevalência de IST como desfecho das mulheres privadas de liberdade da Cadeia Pública Feminina de Boa Vista, Roraima, 2017.

Variável explicativa	OR ajustado	IC95%	p*
Ter mais de 30 anos	2,57	1,03–6,40	0,04
Escolaridade até ensino fundamental	2,77	1,08–5,05	0,03
Pouco conhecimento sobre o uso da camisinha	2,37	1,01–7,31	0,04
Acha não ter risco de pegar sífilis	2,36	1,08–6,50	0,03
Abuso sexual	4,93	0,48–49,8	NS
Não sabe do HIV	1,88	0,71–5,08	NS
Uso da cocaína	2,72	0,31–23,4	NS
Acha não ter risco de pegar hepatite B	0,87	0,67–1,45	NS

* O nível de significância estatística foi fixado em p < 0,05.

NS: não significativo

## DISCUSSÃO

O Brasil possui a terceira maior população carcerária do mundo – em termos absolutos, perde apenas para os Estados Unidos e a China ^11^ –, e o entendimento da epidemiologia dos principais agravos dessa população, sobretudo das doenças infectocontagiosas, ganha notória relevância em saúde pública. Este foi o primeiro estudo a analisar a epidemiologia de HIV/IST em detentas em Roraima, ressaltando-se sua elevada taxa de participação (93,8% da população feminina privada de liberdade). Uma alta prevalência de IST foi observada em nossa amostra. Uma em cada cinco mulheres que cumprem pena na Cadeia Pública Feminina de Boa Vista apresentou resultado positivo no teste rápido de alguma IST – prevalência maior que a relatada em estudo semelhante realizado no Ceará em 2010, com 155 detentas, no qual 13,5% das mulheres apresentaram alguma IST ^12^ . Para compreensão da magnitude da prevalência de IST em pessoas privadas de liberdade, vale a comparação com resultados de um estudo realizado com a população geral de Roraima. Fonseca et al. ^13^ fizeram um inquérito domiciliar de soroprevalência de IST em Roraima em 2017. Dos 727 adultos pesquisados, 5,8% apresentaram alguma IST, porcentagem significativamente menor que na população privada de liberdade do mesmo estado. Considerando apenas as mulheres incluídas na mesma pesquisa (n = 420), a prevalência de IST foi de 5,5%.

Quanto ao HIV, a prevalência de infecção no presídio (4,7%) foi cinco vezes maior que na população geral de Roraima (0,9%) ^13^ . Embora estudos em populações carcerárias sejam escassos, alguns reportaram, nas décadas de 1980 e 1990, elevadas prevalências de infecção por HIV em prisões femininas no Brasil – em São Paulo, por exemplo, Queiroz et al. ^14^ encontraram taxa de 18,3% em 1987. Até o limite de nosso conhecimento, a maior prevalência de infecção por HIV já descrita em presídios femininos brasileiros foi de 26%, também em São Paulo, em 1997 ^5^ . Estudos mais recentes sugerem uma tendência à redução da prevalência de infecção por HIV em presídios femininos nas últimas décadas no Brasil. Em 2012, no Mato Grosso do Sul, a prevalência dessa infecção foi de apenas 0,7% ^15^ , e em estudo realizado em 12 presídios de cidades da região Centro-Oeste, em 2014, foi de 1,9% ^16^ . Neves et al. ^17^ descreveram 4,9% de positividade do HIV em presídio feminino de Goiás, dado semelhante ao encontrado em Roraima. Já em estudo de âmbito nacional sobre o perfil da população penitenciária feminina brasileira em 15 presídios (de nove estados), realizado nos anos de 2015 e 2016, foi relatada prevalência de 2,3% ^18^ .

A prevalência de sífilis encontrada neste estudo foi quase cinco vezes maior que a da população geral de Roraima (3,2%) ^13^ e quase três vezes superior às estimativas de prevalência nacional (5,2%) ^19^ . Embora muito elevada, essa taxa ainda é menor que a de mulheres privadas de liberdade em Recife (PE), em 2013 (23,8%) ^20^ , e em São Paulo, em 2000 (22,8%) ^7^ . Porém, é similar à prevalência encontrada por Miranda et al. ^21^ no Espírito Santo, que era de 15,5% em 1997. A ausência de marcador de infecção pelo vírus da hepatite B (VHB) na população estudada chamou muito a atenção, pois a infecção pelo VHB apresenta rotas de transmissão similares às do HIV, sendo comum a coinfecção. Além disso, as evidências apontam elevada prevalência do VHB na população privada de liberdade, como em presídios de Goiânia (GO) (26,4%) e de Ribeirão Preto (SP) (19,5%) ^8 , 22^ . Uma expressiva parcela das mulheres estudadas acredita não correr risco de contrair hepatite B, a ponto de ser significativa estatisticamente a chance de infecção – o que se pode considerar, portanto, um risco futuro de infecção pelo VHB. Vale ressaltar que desconhecemos a situação vacinal dessas mulheres.

A maioria das participantes do presente estudo possuía baixa escolaridade, corroborando evidências da literatura. No estudo de Freire ^18^ , 61,4% da população de 15 penitenciárias femininas em nove estados brasileiros não tinha acessado o ensino de nível médio. Dados nacionais recentes mostram que 65% da população prisional feminina brasileira ainda não acessou o ensino médio, tendo concluído, no máximo, o nível fundamental ^10^ . A baixa escolaridade foi uma das variáveis com maior associação com IST em nossa amostra, triplicando a chance de infecção. Camargo et al. ^23^ relataram que a maioria das presidiárias portadoras de HIV não haviam terminado o primeiro grau. O baixo nível de instrução dificulta o acesso e o entendimento das informações sobre IST, limitando a adoção de comportamento preventivo ^24^ . O abandono precoce dos estudos em virtude de envolvimento com atividades ilícitas pode contribuir para esse fenômeno na população feminina privada de liberdade. Ressalta-se, portanto, a vulnerabilidade desse grupo num contexto de pauperização da epidemia da aids ^15^ .

Ainda nesse sentido, um terço das detentas que informaram desconhecer a aids tiveram resultado positivo de alguma IST, e esse desconhecimento quase triplicou a prevalência de infecção. Kuznetzova et al. ^25^ , ao analisar o conhecimento de detentas sobre HIV/aids, descreveram inúmeros equívocos que alimentavam falsas crenças sobre as formas de prevenção e transmissão da doença. Estudos comportamentais confirmam que a atitude é modulada por diversos fatores, como crenças, emoções e valores; porém, a influência do conhecimento no comportamento é fundamental, de forma positiva ou negativa ^26^ .

Quanto à percepção sobre IST, apesar de distorções terem sido frequentes, não se correlacionaram fortemente com a chance de infecção neste estudo, à exceção da autopercepção de invulnerabilidade. A sensação de não correr risco de contrair sífilis e hepatite B mais que duplicou a chance de infecção. Portanto, as correlações entre desconhecimento, percepções distorcidas e positividade de IST evidenciadas em nosso estudo validam as observações de Pelto e Pelto ^27^: atitudes causais ou equivocadas podem ser consideradas produto de crenças ou desconhecimento. O mesmo pode ser interpretado quanto ao desconhecimento sobre o uso da camisinha. Aproximadamente 20% da amostra não sabia que o uso de preservativos previne a transmissão de HIV, sífilis e hepatite B. A prevalência de infecção foi significativamente superior em mulheres com déficits de conhecimento sobre a principal forma de prevenção das IST. A Organização Mundial da Saúde (OMS) recomenda, no guia sobre infecção de HIV/aids na prisão, que tanto os presos quanto os profissionais que trabalham no sistema prisional devem receber educação continuada acerca da prevenção e transmissão do HIV ^28^ . Na maior parte dos presídios do Brasil são raras as ações de educação em saúde direcionadas ao controle das IST. A realidade da saúde em prisões ainda é precária e negligenciada ^29^ .

Como era esperado, o uso de drogas injetáveis se correlacionou às IST em nossa amostra, sugerindo que o compartilhamento de seringas é um vetor importante nesse grupo, seja antes ou durante o cárcere. Há evidências de que a exposição a comportamentos de risco dos sujeitos privados de liberdade costuma se iniciar ainda quando são indivíduos livres, antes do encarceramento ^15^ . Entretanto, uma variável chamou a atenção, tanto por ter apresentado correlação mais forte com a chance de ter IST quanto por demonstrar a vulnerabilidade dessas mulheres durante seu aprisionamento: 8,3% delas referiram ter sofrido abuso sexual na prisão, e metade destas tiveram resultado positivo nos testes de IST, o que representa uma chance quase cinco vezes maior de infecção em comparação àquelas sem relato de abuso. Superlotação, exposição a violência física, deficiência de iluminação, restrição de acesso à Justiça e a serviços médicos contribuem para o aumento da vulnerabilidade dessa população às IST, a exemplo do risco elevado de abuso sexual dentro dos muros da prisão ^30^ . Essa situação reflete um grave problema de saúde pública, pois o sistema prisional pode funcionar como ambiente propício à disseminação de IST, como local de aglomeração dessas infecções e, por fim, como foco disseminador para a população em geral ^31^ .

Apontamos como limitação deste estudo a restrição da avaliação das IST a HIV, sífilis e hepatite B, não tendo sido consideradas as demais infecções. Também não foram abordados aspectos comportamentais, como o consumo de álcool, e, adicionalmente, é possível ter havido viés de resposta, apesar de todos os esforços dos pesquisadores para obter informações válidas, pois foram abordados assuntos delicados relativos a comportamento de risco.

A população privada de liberdade constitui um grupo de alta vulnerabilidade às IST. A elevada prevalência dessas infecções pode ser explicada por déficits de conhecimento sobre o assunto, percepções distorcidas e condições peculiares ao aprisionamento, que resultam em comportamento de risco. Ressalta-se a necessidade de implantação de programas educativos de prevenção, diagnóstico e tratamento de IST para essa população. Consideramos importante a expansão do estudo para outras infecções, bem como um estudo similar para a população masculina privada de liberdade do estado de Roraima.
